# Physical activity: associations with health and summary of guidelines

**DOI:** 10.29219/fnr.v67.9719

**Published:** 2023-06-26

**Authors:** Katja Borodulin, Sigmund Anderssen

**Affiliations:** 1 Age Institute, Helsinki, Finland; 2 Department of Sports Medicine, Norwegian School of Sport Sciences, Oslo, Norway

**Keywords:** guidelines, health, physical activity, population, sedentary behavior

## Abstract

The understanding of how physical activity and insufficient physical activity are associated with health outcomes has increased considerably over the past decades. Along with physical activity, the evidence on the associations between sedentary behavior and health has increased, which has resulted in the introduction of recommendations of sedentary behavior. In this article, we 1) present terminology for physical activity and sedentary behavior epidemiology, 2) show the relevant scientific evidence on associations of physical activity and sedentary behavior with selected health-related outcomes and 3) introduce the global guidelines for physical activity and sedentary behavior by the World Health Organization (WHO). Health-related outcomes include cardiovascular morbidity and mortality, total mortality, glucose regulation and type 2 diabetes, adiposity, overweight, obesity, cancer, musculoskeletal and bone health, brain health, and quality of life. These health-related outcomes are reflected across age groups and some population groups, such as pregnant and postpartum women. Furthermore, we discuss physical activity levels across Nordic countries and over time. For the Nordic Nutrition Recommendations, shared common physical activity guidelines were not developed. Instead, each country has created their own guidelines that are being referenced in the article, along with the global WHO guidelines.

## Popular scientific summary

- The understanding of how physical activity and sedentary behavior are associated with health outcomes increases over time when more research on disease-specific outcomes, age groups, and special population groups evolves. With new evidence, guidelines on physical activity and sedentary behavior are updated. We present associations of physical activity and sedentary behavior with health-related outcomes, the updated guidelines by World Health Organization (WHO) and give references to the country-specific guidelines in the Nordic and Baltic countries.

The understanding of how physical activity and insufficient physical activity are associated with health outcomes has increased considerably over the past decades. Epidemiologic research, clinical interventions, and mechanistic studies have contributed to the evidence that physical activity is essential to preventing disease, improving health, and improving quality of life. Physical activity can be done in different domains such as during leisure time, education, occupation, and transportation. The reference list in this chapter includes several key references but does not intend to cover the entire body of literature regarding the effects of physical activity on health. Literature search for this chapter relied on recent systematic literature search processes that were carried out by many national guideline development processes and the WHO guideline development processes. The most recent update on existing literature was updated in the WHO process, including literature published until September 2019. Thus, in this chapter we use the most recent reviews, including umbrella reviews as well as some selected articles on each disease group.

Healthy diet alone or sufficient physical activity alone have a huge impact on our health and wellbeing. However, since there is a strong interaction between nutrition and physical activity they cannot be totally separated when making recommendations for either one of them. For a good public health impact, a combination of healthy diet and sufficient physical activity is needed.

## Glossary

*Aerobic physical activity is activity that increases heart rate and breathing, involves large muscles in repetitive movements in a sustained period of time. Also known as endurance training. Examples are walking, jogging, bicycling, skiing and swimming.*

*Balance training improves an individual’s ability to sustain postural balance and prevent falling in spite of postural sway or stimuli from self-motion, the environment or other objects. Examples are static or dynamic exercises that challenge body’s center of gravity, such as dance and gymnastics.*

*Bone-strengthening activity increases the strength of specific sites in bones that make up the skeletal system. Movements that produce impact or tension force on the bones lead to bone growth and strength. Examples are hopping, running, gymnastics, lifting weights, and racket games.*

*Domains of physical activity refer to context where activity takes place, such as leisure-time, occupation, education, household, or transportation.*

*Endurance training is repetitive, dynamic use of large muscles (e.g. swimming, walking, or bicycling).*

*Exercise is any planned, structured, and repetitive bodily movement carried out to improve or maintain one or more components of physical fitness.*

*Household domain physical activity is performed in the home including domestic tasks like cleaning, childcare, gardening or snow shoveling.*

*Leisure-domain physical activity refers to activities like sports participation, exercise conditioning or training, and recreational activities like walking, dancing and gardening.*

*Light intensity activity is defined as activity corresponding to an energy expenditure between 1.5 and 3 metabolic equivalent of tasks (MET) such as standing or walking slowly (<3.5 km/h).*

*Major muscle groups are legs, back, abdomen, shoulders and arms.*

*Metabolic equivalent of task is a unit used to estimate energy expenditure (oxygen consumption) of physical activity. One MET equals energy expenditure at rest and corresponds to approximately 3.5 mL O2·kg^-1^·min^-1^.*

*Moderate intensity physical activity is defined as activity that requires three to six METs.*

*Multicomponent physical activity refers to activities that combine elements of aerobic, strength, balance, agility or flexibility training. Multicomponent activity is targeted to older adults to prevent falling and maintain mobility. Examples are stair climbing, weightlifting, gymnastics, and dancing.*

*Muscle-strengthening activity is exercise designed to increase skeletal muscle strength, power, endurance and mass. Examples include strength or resistance training.*

*Occupation domain physical activity is undertaken during work, which can be paid or voluntary working.*

*Physical activity is a comprehensive concept that encompasses many terms related to movement of the body. It is defined as any bodily movement achieved by contraction of skeletal muscles that increases energy expenditure (EE) above resting levels.*

*Physical fitness is a set of attributes related to the ability to perform physical activity and is something that people ‘have’ or ‘strive to achieve’. The term includes cardiorespiratory fitness, strength, coordination, flexibility, etc.*

*Physical inactivity is insufficient physical activity and is defined as a failure to meet the current recommendations.*

*Sedentary behavior refers to any waking activity characterized by an energy expenditure ≤ 1.5 metabolic equivalents and a sitting or reclining posture. In general, this means that any time a person is sitting or lying down they are engaging in sedentary behavior. Common sedentary behaviors include TV viewing, video game playing, computer use (collectively termed ‘screen time’), driving automobiles, and reading.*

*Transport domain physical activity is performed to get from one place to another in physically demanding modes, such as walking, bicycling or wheeling. Term active transport is also used.*

*Vigorous intensity physical activity is activity requiring more than 6 METs.*

## Study designs and measurement challenges in physical activity

Health benefits of physical activity are broadly reported across different population groups and across different health outcomes. Much of the existing evidence relies on observational studies, such as cohort studies that have followed participants over time after baseline measurements or cross-sectional observations with physical activity and health indicator being measured at the same time. However, evidence is also available from randomized clinical trials, where the causality from physical activity as an exposure can be estimated with the outcome indicator in a more controlled way than in observational studies. Examples from observational studies include outcomes such as cancer and cardiovascular diseases, and from clinical trials outcomes like gait speed, osteoarthritis, osteoporosis and diabetes type 2. Measurement of physical activity has shown to be a challenge, as there is no gold standard for self-reported methods. Laboratory measurements cannot be carried out for large samples, and movement device-based methods have their own challenges (1). However, movement devices, such as accelerometry have recently become more broadly used, also in large-scale cohort studies.

The guidelines are largely based on the information gained from studies reporting self-reported physical activity behavior, but device-based information adds to this body of evidence where appropriate.

## Morbidity and mortality from cardiovascular diseases and all-cause mortality

Physical activity of any intensity is shown to associate with all-cause and cardiovascular disease mortality in numerous studies (2). It is estimated that physical inactivity may account for 9% of premature mortality (3). Leisure-time physical activity may bring from 1.9 to 2.4 additional life-years in men and from 1.5 to 1.8 life-years in women when comparing groups of no leisure-time physical activity with low to high volume of leisure time physical activity (4).

Previous prospective studies, systematic reviews and meta-analyses have shown inverse associations of physical activity with all-cause mortality (5–10) and with cardiovascular disease incidence and mortality (3, 7, 8). A recent meta-analysis (6) in adults using a median follow-up of 5.8 years showed a dose-response association between total accelerometer-based physical activity and all-cause mortality. The mortality risk, as compared to the least active 1^st^ quartile, was 46% lower in 2^nd^ quartile, 59% lower in 3^rd^ quartile, and 66% Iower in 4^th^ quartile. Similar inverse associations were found for light intensity physical activity and for moderate-to-vigorous intensity physical activity. The greatest risk reductions for mortality were seen at 375 min/d of light-intensity physical activity or 24 min/d of moderate-to-vigorous intensity. Another meta-analysis (8) concluded that reaching recommended level of physical activity 750 MET/min week was associated with a 14% lower risk of all-cause mortality and a 27% lower risk of cardiovascular mortality, when compared to those not reaching the recommended level of physical activity.

Low intensities of physical activity are suggested to associate with cardiovascular disease and all-cause mortality (6), but there are also studies suggesting weak or no association (7,11). It is likely that studies differ in their representativeness, age range, loss to and length of follow-up, and placement and accuracy of devices that may all have a role in the inconsistent findings.

Daily steps are seen as an option for future recommendations as steps can be understood by lay people and are easily quantified using simple device. Daily steps are reported to inversely associate with several health outcomes (12). It is suggested that every 1000 increase in daily step count is associated with a 6-36% lower risk for all-cause mortality and a 5%–21% lower risk for cardiovascular events (13). Furthermore, in a cohort of older women (14), it was shown that compared to daily step count of 2700, already 4400 daily steps were associated with a lower mortality risk and that the risk gradually decreased until 7500 steps.

Meeting the guidelines for both aerobic and muscle strengthening activities and meeting just muscle strengthening activities showed 29% and 20% lower all-cause mortality risk, respectively when compared to those not meeting those guidelines (15).

Different domains of physical activity have also been studied for health benefits. A review (16) of 11 prospective cohorts, albeit with large heterogeneity, concluded that active commuters had 8% lower all-cause mortality risk in comparison to inactive persons. For occupational physical activity, associations with cardiovascular or all-cause mortality have shown mixed findings and methodological shortcomings such as heterogeneity in the classification of occupational physical activity and residual confounding from socioeconomic factors (17–19). A meta-analysis suggested an 18% higher risk of all-cause mortality in men with high occupational physical activity compared to those with low occupational activity (17). Dalene et al (2021) suggested a positive dose-response relationship between occupational physical activity and longevity in men (18). Another meta-analysis (19) found occupational physical activity not to associate with overall cardiovascular diseases, but to associate directly with a 15% increase in ischemic heart disease mortality risk.

Sedentary behavior is suggested to associate, independent of physical activity, with cardiovascular disease incidence and mortality (20–22), as well as with all-cause mortality (6, 7, 20, 21). Ekelund et al (6) suggest hazard ratios of 1.28 in 2^nd^ quartile, 1.71 in 3^rd^ quartile, and 2.63 in 4^th^ quartile, as compared to the least sedentary quartile 1, in which the least sedentary people spent 7.5–9 h/day (accelerometry-based). Some large cohort studies, however, have also reported non-significant associations between sedentary behavior and cardiovascular disease (7, 11). Independent associations are reported between TV time and all-cause and cardiovascular mortality (21). However, studies have also pointed out that the detrimental effects of sedentary behavior can be attenuated or even prevented by physical activity (8,9, 23–25). Reaching the upper limit of physical activity recommendation can outweigh the harms of sedentary behavior (23).

A literature review that was carried out for the update of the WHO 2020 guidelines came into the conclusion that current evidence does not allow quantifying the cut-off points for recommended time in sedentary behavior, nor is there enough evidence to make specific recommendations on the type or domain of sedentary behavior, or frequency or duration of bouts or breaks in sedentary behavior (20).

## Glucose regulation and type 2 diabetes

The evidence from prospective cohort studies and from randomized control trials show inverse associations between physical activity and type 2 diabetes or its pre-clinical conditions, including elevated blood glucose levels (26). The population attributable fraction from low physical activity is estimated to be 7% for type 2 diabetes (3).

A recent systematic review summarized evidence on associations between accelerometry-based daily step counts and dysglycemia from eight prospective studies that had a follow-up time from 3 months to 5 years (13). Outcome on dysglycemia included elevated blood glucose levels and HbA1c, insulin resistance, 2-h glucose, insulin sensitivity and incident dysglycemia or type 2 diabetes. Their findings suggest mixed results, where non-significant or weak inverse associations were found. Two studies showed 2% and 13% lower diabetes and incident dysglycemia risk for each 1000-steps and 2000-steps increase, respectively.

Sedentary behavior may increase the risk of type 2 diabetes, independent of physical activity, as found in a systematic review and a meta-analysis using 11 prospective studies (21). A relative risk of 1.01 in total sitting time and 1.09 in TV viewing time were found for type 2 diabetes and a population attributable fraction of 29% for TV viewing. Similar associations were observed from another systematic review that found 11% higher risk of incident type 2 diabetes with higher level of sitting time (27). When reallocating 30 min of sedentary behavior in substitution analyses to light intensity activity, beneficial associations were suggested for fasting insulin, and when reallocating to moderate to vigorous physical activity, even stronger associations were suggested to fasting glucose and insulin (28).

## Adiposity, overweight and obesity

Physical activity is associated with maintenance of healthy weight and attenuation of weight gain in adults (29–31) and with reduction of excessive increase in body weight and adiposity in children (9, 32, 33). Moderate-to-vigorous intensity physical activity has been shown to associate with adult-age prevention of weight gain and the association may be even more pronounced when exceeding 150 min/week of moderate-to-vigorous intensity physical activity (34). Also, combining dietary restrictions and physical activity shows to be effective in weight loss (35).

The evidence of the association between physical activity and adiposity is unsystematic and heterogenous, despite the large amount of research on this topic (9). Therefore, the strength of evidence in most recent reviews and guidelines has been stated as limited or not assignable, where many research gaps are related to dose-response-associations and specific types of physical activity (9, 36). Furthermore, research gaps are recognized for associations of physical activity or sedentary behavior with sociodemographic variables and ethnicity (36).

Concerning sedentary behavior, the level of evidence on health outcomes is weaker than evidence found for physical activity. From existing systematic reviews and meta-analyses, low certainty evidence suggests that time spent in sedentary activities may have a role in different measures of adiposity and weight status in school-aged children (9, 37) and in adults (20, 38, 39). There is limited evidence available on associations of different types of sedentary behavior with adiposity (40).

Replacing 30 min of daily sedentary time with light intensity physical activity was found to be associated with reductions in waist circumference (28). In the same meta-analysis, replacing sedentary behavior with moderate-to-vigorous intensity physical activity showed even larger effect on reducing waist circumference and body mass index.

## Causal pathways concerning cardiovascular disease, glucose regulation and adiposity

Non-communicable diseases progress through life, and the biological mechanisms are complex. The causal pathways from physical activity or sedentary behavior to cardiometabolic health outcomes share similarities for cardiovascular disease, type 2 diabetes, and obesity. Physical activity has favorable effects on cardiometabolic health, particularly by lowering the risk factor levels for blood pressure, metabolic syndrome, type 2 diabetes, and blood fatty acids and facilitates glucose homeostasis (3, 41). Physical activity improves risk factor levels through a role in low grade inflammation. As importantly, people with diagnosed cardiovascular diseases can postpone the progression of the disease by engaging in physical activity (9). For sedentary behavior, the causal pathway is suggested to be the opposite to physical activity. Sedentary behavior may increase all relevant metabolic risk factors for cardiovascular disease and subsequently lead to incident cardiovascular disease.

Potential mechanisms from the benefits of physical activity on glucose regulation are well known (36). Being physically active increases body metabolism in multiple ways and has direct effects on circulating glucose levels, subsequently on insulin resistance, and energy consumption. Physical activity may also prevent abdominal obesity and reduce subcutaneous fat, thus acting as a mediating factor between obesity and glucose irregulation.

Physical activity is essentially part of energy consumption and directly affects whether body energy balance is negative or positive. The causal pathway from physical activity or sedentary behavior is assumed to affect through increased metabolism and increased energy uptake. Biological mechanism is complex, as physical activity may alter body composition such as muscle or fat mass, while body weight remains unchanged. It is likely that physical activity brings health benefits regardless of the adiposity level, which is often referred to the ‘fit but fat’ theory (42, 43). Physical activity, sedentary behavior, and adiposity jointly account for prevention of important major diseases like cardiovascular disease and type 2 diabetes.

## Cancer

A systematic review from 45 studies suggests a strong association between physical activity and bladder, breast, colon, endometrial, esophageal adenocarcinoma, renal, and gastric cancers (44). The relative risk reduction for these cancer types varied from 10%–20% between highest and lowest physical activity categories. Similar associations are reported by the WHO 2020 Guidelines Development Group (9) and the 2018 Physical Activity Guidelines Advisory Committee (36), where it is stated that the evidence is insufficient concerning the associations between physical activity and hematologic, head and neck, ovary, pancreas, prostate, thyroid, rectal and brain cancer. Lung cancer is largely confounded by tobacco use.

Physical activity may also play a role in post-diagnosis survival rate (44, 45), as two systematic reviews found moderate or limited associations between physical activity and decreased all-cause and cancer-specific mortality in individuals with a diagnosis of breast, colorectal, or prostate cancer, where relative risks varied from 40% to 50%.

For sedentary behavior, moderate level evidence is reported for the associations between sedentary behavior and incident endometrial, colon and lung cancer, while limited evidence was found on associations for cancer mortality (39).

The required dose of physical activity needed for a lower risk of cancer varies between studies, although some evidence on dose-response-type of associations has been suggested (44). This has also been recognized for sedentary behavior (20). The type of physical activity or sedentary behavior is still an area where more research needs to be done to understand the associations between type of activity and cancer risk (20). The available evidence on cancers has been shown in adult populations, but separate groups such as sex, ethnicity, and weight status have been studied sparsely and have covered selected site-specific cancers.

Causal pathways for associations between physical activity, sedentary behavior and cancer prevention are largely suggested through metabolic processes. These processes are seen as mechanistic, hypothesized models, as carcinogenesis is a long process and difficult to show in typically used study designs in humans (46). It is suggested that pathways from physical activity to lower cancer risk are related to sex hormones, metabolic hormones, inflammation and adiposity, immune function, oxidative stress, DNA repair, and xenobiotic enzyme systems (46, 47). This evidence is gathered from studies using many designs, such as randomized controlled trials, cross-sectional, cohort and case-control studies, as well as animal models. Furthermore, the benefits of exercise on cancer treatment and on post-treatment wellbeing may act through improved physical fitness, maintained muscle and bone mass and cardiac rehabilitation (46).

## Musculo-skeletal and bone health

Physical activity and diet are the primary modifiable risk factors associated with bone health (48, 49). Optimization of lifestyle factors, shown to influence 20%–40% of adult peak bone mass, is important to reduce osteoporosis later in life. Physical activity, adequate intake of calcium and vitamin D as well as stratification of fracture risk should be the main targets to prevent osteoporosis and fractures (48). Reversible risk factors for falls include weak lower limb muscle strength, poor balance, and a poor level of overall physical fitness, all of which can be improved by regular physical activity (50). Muscle strength and muscle endurance diminish with increasing age and decreasing activity level, and physical activity can counteract and reverse this trend to a substantial degree.

Physical activity contributes to increased bone density and can counteract osteoporosis, and physical activity immediately before and during puberty seems to yield greater maximum bone density in adult life. In women both before and after menopause and in middle-aged and older men, a beneficial effect on bone density has been shown. The evidence is based on systematic reviews and meta-analysis (51–54). However, there is a need to further explore possible gender differences with respect to the effect of exercise on bone health. To be beneficial for bone mass and structure, exercise should preferably be weight-bearing, and repeated weight-bearing and loading, such as walking and running, is more beneficial than activities such as swimming and cycling. Even better for bone health are activities with high impacts (e.g. tennis, squash, and aerobics) or high-volume loading (weight training). Information about the dose-response relationship between physical activity and osteoporosis is not conclusive enough and warrants future research. Possible mechanisms of physical activity are beneficial influence of the balance between osteocytes and osteoblasts, and on hormones acting on the skeleton (for instance growth hormone and IGF-1) (55).

Osteoarthritis is also a prevalent disease where physical activity and healthy weight are significant for musculoskeletal health. With increasing inactivity and obesity, the prevalence of osteoarthritis has also increased significantly, also in the middle-aged population. The Physical Activity Guidelines Advisory Committee (PAGAC) investigated seven chronic conditions, among them osteoarthritis (36). Osteoarthritis affects a large portion of the general population (13.4% of the adult US population and 14% of the Norwegian population above 20 years of age) and is associated with high disability (56). There is high quality evidence that physical activity and exercise are effective for people with osteoarthritis (57). Physical activity and exercises that amount up to those consistent with 150 min/week of moderate-intensity have a substantial beneficial impact on health of individuals with osteoarthritis (57).

We have high quality evidence that joint injury, obesity and muscle weakness are modifiable risk factors for osteoarthritis. Early risk-based interventions are highlighted as significant for primary and secondary prevention of osteoarthritis (58).

## Brain health

There is evidence that regular physical activity reduces the risk of developing anxiety and depression (59). In a meta-analysis including more than 250 000 individuals around the world, it was shown that individuals with high levels of physical activity had lower likelihood of developing depression compared to those with low levels of physical activity. This was true in youth, adults and the elderly, and protective effects against depression were found regardless of geographical region (60). However, there is not enough data to determine dose-response relationships between physical activity and depression and anxiety. There is also evidence that both acute and regular physical activity can influence quality of life and sleep (36). There is evidence supporting the hypothesis that physical activity can slow down the progression of Alzheimer’s disease (61). Also, increased amount of physical activity is associated with improvement of brain function and structure, and cognition. Evidence suggests that the greatest effect is on executive function and memory. The positive effects of physical activity herein seemed to be independent of the type of activity. The mechanism is largely unknown; however, regular physical activity may have an impact on the creation of neurons and new blood vessels in the brain. Moreover, physical activity may have a beneficial effect on inflammatory markers (62).

Further research is needed to study the volume and mode of physical activity that is most beneficial to brain health (cognition, mental health and quality of life), and to explore the mechanisms through which physical activity improves cognition. Further studies should also include sedentary behaviors as an exposure.

## Population group-specific conditions

### Children and adolescents

Regular physical activity is necessary for normal growth and the development of cardiorespiratory endurance, muscle strength, flexibility, motor skills, cognitive function, academic outcomes and agility (2, 33). In addition, physical activity during the formative years strengthens the bones and connective tissues and yields greater maximum bone density in adult life. Physical activity that provides high impact loading on bones is important for bone development, particularly during early puberty (2). There is also evidence of an association between physical activity and cardiovascular disease risk factors in children and adolescents (63). Furthermore, risk factors such as fatness, insulin glucose ratio, and lipids tend to cluster in children and adolescents with low cardiorespiratory fitness and low levels of physical activity (63, 64). There is a growing body of evidence of a favorable association between physical activity and fundamental motor skill development and academic performance in children (65, 66). Furthermore, children and adolescents who are involved in physical activity seem to experience fewer mental health problems (2). There is no indication that increased physical activity in school represents any risk of impairing children’s cognitive skills as a result of less time for theoretical school subjects (67).

For children of all ages, the associations between sedentary behavior and health outcomes are in line with the information given in the earlier sections. Relevant issues in children are related to motor skill development, sleep, academic achievements, and social interaction, for which evidence suggests inverse associations against sedentary behavior (2, 33). Furthermore, unfavorable associations of sedentary behavior with well-being and quality of life are noted in school-aged children and adolescents (2). Moreover, in this group, higher durations of screen time, television viewing and video game use may be associated with poorer mental health and pro-social behavior in children and adolescents (2).

### Older adults

For older adults (referring to people aged 65 years and above), all of the health outcomes from physical activity and sedentary behavior apply as they are for any adults. Furthermore, association of physical activity or sedentary behavior with functional capacity and risk of falls and fall-related outcomes are particularly relevant in the older population.

Systematic reviews and a broad body of evidence show that physical activity associates with and improves physical function (9,68–71). Aerobic, muscle-strengthening and multicomponent physical activity programs show the largest improvements in functional capacity (68). Furthermore, physical activity is suggested to associate with better mobility, and a decline in physical activity to decrease life-space mobility and to increase a risk to develop a walking difficulty (72). A large cohort of community-dwelling older people using accelerometry-based physical activity and sedentary time suggested that higher moderate-to-vigorous intensity physical activity was associated with better hand grip strength, faster usual walking speed and faster timed chair stand speed (73). No associations, independent of moderate-to-vigorous intensity physical activity, were found between sedentary behavior and functioning (73). There is increasingly more evidence showing that people with physical impairments and mobility decline benefit more from exercise training than people with less functional impairments (70).

Fall prevention is one of the relevant outcomes that have recently evolved new evidence. It has been shown in randomized controlled trials that exercise reduces the rate of falls by 23% (74). Balance and functional exercises, as compared to control, showed a 24% decrease in the rate of falls in 39 studies and further a 42% reduction in rate of falls if the weekly dose of training exceeded 3 h (74).

### Pregnant and postpartum women

Aerobic and muscle strengthening physical activity is recommended for women with uncomplicated pregnancies before, during and after pregnancy, although some modifications to exercise routines might be needed due to normal anatomic and physiologic changes and fetal requirements (75).

Physical activity brings benefits to pregnant and postpartum women, where systematic evidence is shown in the prevention of gestational weight gain (76–78) and gestational diabetes mellitus (78–80), also covering physical activity before pregnancy and women with overweight and obesity. Weight gain is reported to be 1.14 kg lower among pregnant women when physically active are compared to physically inactive, and the risk for gestational diabetes was 29% lower for the active women. The needed dose has varied across existing studies, but the recommendation of 150 min/week of moderate intensity physical activity has often been used.

Furthermore, it is shown that physical activity does not increase the likelihood for gestational hypertension or preeclampsia (78, 79), does not increase the risk of adverse effects, such as those on fetal outcomes (77–85) or delivery complications, including pre-term birth and birthweight (82). There is some evidence suggesting that physical activity during pregnancy may be protective against low birthweight, also in overweight and obese women or large-for-gestational-age babies (77). For mental health outcomes, it is demonstrated that physical activity during pregnancy may be inversely associated with postpartum depression (85).

Pregnancy and childbirth bring challenges to the musculoskeletal system, especially to the pelvic floor, lower back, pelvis and abdominal area. These challenges may decrease women’s ability to participate in physical activity. Continent women who do pelvic floor muscle training during pregnancy are 62% less likely to experience urinary incontinency in late pregnancy and 29% less in postpartum (86). Furthermore, postpartum pelvic floor muscle training can reduce urinary incontinence (86).

For sedentary behavior, the research covering pregnancy and postpartum has been scarce. While sedentary behavior has been shown to associate with many adverse health outcomes in the adult population, this protective mechanism may also apply to pregnant and postpartum women. The causal pathways from physical activity or sedentary behavior to health outcomes are similar to those described earlier in this report.

## The WHO recommendations on physical activity and sedentary behavior

The most recent guidelines for physical activity and sedentary behavior were launched by WHO (2, 33) as given in detail in Tables 1 and 2 below. The guidelines were developed in accordance with the WHO Handbook for guideline development. The guideline development group defined critical and relevant health outcomes, including both benefits and harms, and created PI/ECO (Population, Intervention/Exposure, Comparison, Outcome) questions that served the process for evidence evaluation. Systematic reviews of evidence for critical and important health outcomes were performed by external reviewers and rated according to AMSTAR 2 (Assessment of Multiple Systematic Reviews) instrument. The evidence was rated from high to critically low, stating the quality of available studies. Furthermore, the body of evidence was synthesized using GRADE (The Grading of Recommendations Assessment, Development and Evaluation) method for each PI/ECO question. The GRADE rating reflected the certainty of evidence, ranging from high to very low. After the process of evaluating the available evidence, the guideline development group synthesized the body of evidence into recommendations, separately for physical activity and sedentary behavior, as well as for separate age and population groups. Each of the recommendation was graded strong, limited or not assignable (Tables 1 and 2). The guidelines underwent an international public consultation round before their launch.

**Table 1:** World Health Organization guidelines on physical activity, sedentary behavior and sleep for children under 5 years of age (33)

Population group	Physical activity guidelines	Sedentary behavior guidelines	Sleep
**Children under 5 years of age**	In a 24-h day; Infants under 1 year should have each day at least 30 min of physical activity; Children aged 1–2 years should do at least 180 min of physical activity; Children aged 3–4 years should do at least 180 min of physical activity, of which at least 60 min moderate to vigorous intensity physical activity. (strong recommendations, very low quality evidence)	In a 24-h day; Infants under 1 year should not be restrained for more than 1 h at a time. Screen time is not recommended; Children aged 1–2 years should not be restrained for more than 1 hour at a time. For 1-year-old children, sedentary screen time is not recommended. For those aged 2 years, sedentary screen time is limited to max 1 h daily, less is better; Children aged 3–4 years should not be restrained for more than 1 h at a time or sit for extended periods of time. Sedentary screen time should be no more than 1 h, less is better. (strong recommendations, very low quality evidence)	In a 24-h day; Infants under 1 year should have 14–17 h (0–3 months of age) or 12–16 h (4–11 months of age) of good quality sleep, including naps; Children aged 1–2 years should have 11–14 h of good quality sleep, including naps, with regular sleep and wake-up times; Children aged 3–4 years should have 10–13 h of good quality sleep, which may include a nap, with regular sleep and wake-up times. (strong recommendations, very low quality evidence)
	**Integrated recommendations:** For the greatest health benefits, infants, and young children should meet all the recommendations for physical activity, sedentary behavior and sleep in a 24-h period. Replacing restrained or sedentary screen time with more moderate- to vigorous- intensity physical activity, while preserving sleep, can provide additional health benefits. (strong recommendation, very low quality evidence)		

**Table 2:** World Health Organization 2020 guidelines for physical activity and sedentary behavior (WHO 2020)

Population group	Physical activity guidelines	Sedentary behavior guidelines
**Children and adolescents (aged 5–17 years), including those living with disability**	Should do at least an average of 60 min/day of moderate-to-vigorous intensity, mostly aerobic, PA, across the week; Vigorous-intensity aerobic activities, as well as those that strengthen muscle and bone should be incorporated at least 3 days a week. (strong recommendation, moderate certainty evidence)	Children and adolescents should limit the amount of time spent being sedentary, particularly the amount of recreational screen time. (strong recommendation, low certainty evidence)
**Adults (aged 18–64 years), including those with chronic conditions and those living with disability**	All adults should undertake regular physical activity; Adults should do at least 150–300 min of moderate-intensity aerobic PA, or at least 75–150 of vigorous-intensity aerobic PA, or an equivalent combination of moderate-intensity or vigorous-intensity activity throughout the week for substantial health benefits; Adults should also do muscle-strengthening activities at moderate or greater intensity that involve all major muscle groups on 2 or more days a week, as they provide additional health benefits; (strong recommendation, moderate certainty evidence) Adults may increase moderate-intensity aerobic PA to >300 min, or do >150 min of vigorous-intensity aerobic PA, or an equivalent combination of moderate-intensity and vigorous intensity activity throughout the week for additional health benefits (when not contraindicated for those with chronic conditions). (conditional recommendation, moderate certainty evidence)	Adults should limit the amount of time spent being sedentary. Replacing sedentary time with PA of any intensity (including light intensity) provides health benefits; To help reduce the detrimental effects of high levels of SB on health, adults should aim to do more than the recommended levels of MVPA. (strong recommendation, moderate certainty evidence) (For adults with chronic conditions and those living with disability: strong evidence, low certainty evidence)
**Older adults (aged 65 years and older), including those with chronic conditions and those living with disability**	PA recommendation as for adults; As part of their weekly physical activity, older adults should do varied multicomponent PA that emphasizes functional balance and strength training at moderate or greater intensity on 3 or more days a week, to enhance functional capacity and to prevent falls. (strong recommendation, moderate certainty evidence)	SB as for adults.
**Pregnant and postpartum women (see note)**	Undertake regular PA throughout pregnancy and postpartum; Do at least 150 min of moderate-intensity aerobic PA throughout the week for substantial health benefits; incorporate a variety of aerobic and muscle-strengthening activities. Adding gentle stretching may also be beneficial. in addition, women who, before pregnancy, habitually engaged in vigorous-intensity aerobic activity or who were physically active can continue these activities during pregnancy and the postpartum period. (strong recommendation, moderate certainty evidence)	Pregnant and postpartum women should limit the amount of time spent being sedentary. Replacing sedentary time with PA of any intensity (including light intensity) provides health benefits. (strong recommendation, low certainty evidence)

PA = physical activity; SB = sedentary behavior. Additional safety considerations when engaging in PA for pregnant women are as follows: avoid PA during excessive heat especially with high humidity, stay hydrated by drinking water before, during and after PA, avoid participating in activities which involve physical contact, pose a high risk of falling or might limit oxygenation (such as activities at high altitude, when not normally living at altitude), avoid activities in supine position after the first trimester of pregnancy; pregnant women considering athletic competition or exercising significantly above the recommended guidelines should seek supervision from a specialist healthcare provider; pregnant women should be informed by their healthcare provider of the danger signs to stop or limit PA and advised to consult a qualified healthcare providers if they occur. Return to PA gradually after delivery and in consultation with a healthcare provider in the case of cesarean section.

These public health guidelines are for all populations across the age groups, irrespective of gender, cultural background or socioeconomic status and are relevant for people of all abilities. Those with medical conditions and/or disability and pregnant and postpartum women should try to meet these recommendations where possible and as able.

**Fig. 1:**
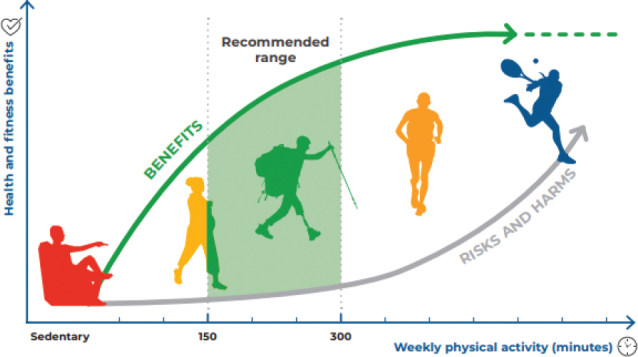
Dose-response curve between physical activity and health benefits (World Health Organization; 2020. Licence: CC BY-NC-SA 3.0 IGO).

It is emphasized that any physical activity is better than none, for all populations groups. For those who are not currently meeting the recommendations, engaging in some physical activity is already health-enhancing (Figure 1). People should gradually increase the frequency, duration, and intensity of physical activity. Furthermore, it is noted that pre-exercise medical clearance is generally unnecessary for individuals without contraindications prior to beginning light-intensity or moderate-intensity physical activity. Adults with chronic conditions can consult a physical activity specialist or health care professional to receive guidance on types and amounts of physical activity based on their needs, abilities, functional limitations, medications and overall treatment plans.

Children and adolescents are to be provided with safe and equitable opportunities and encouragement to participate in physical activity that is appropriate for their age and ability, is enjoyable and offers variety. Older adults are guided to be as active as their functional ability allows and they should adjust the effort of activity relative to their level of fitness.

## Regarding moderate and vigorous physical activity

Examples of energy requirements corresponding to 3–6 METs (moderate intensity activity) and >6 METs (vigorous intensity activity) are given in Table 3. Cardiorespiratory fitness, often expressed as maximal oxygen uptake, decreases as people age. Hence, activity of a certain MET value requires a greater percentage of a person’s cardiorespiratory fitness as he or she ages (see Table 3). Importantly, activity of a certain energy cost might be perceived quite differently even if they are of the same age. For instance, jogging in 8 km/h might be perceived as a light intensity activity for a trained 30-year-old but very hard for an untrained 30-year-old.

**Table 3:** Energy requirements for performing selected various activities in different age groups shown as METs and as percentages of cardiorespiratory fitness (≈ maximal oxygen uptake)

Activities	Energy cost in METs	Energy requirements as percentages of cardiorespiratory fitness (≈ maximal oxygen uptake) and corresponding rating of perceived exertion (Borg scale, in bold) according to age group in years.**			
		Young (20–39)	Middle-aged (40–59)	Old (60–79)	Very old (80+)
Watching TV/reading	1.3	10^<10^	13^<10^	15^<10^	18^<10^
Light household chores	2.5	20^<10^	25 10–11	29^10–11^	35^10–11^
Driving a car	1.5	12^<10^	15^<10^	18^<10^	21^<10^
**Moderate physical activity**					
Climbing stairs	5.5	42^10–11^	55^12–13^	64^14–16^	77^15–17^
Walking (4.8 km/h)	3.5	27^10–11^	35^10–11^	41^10–11^	49^12–13^
Walking (6.4 km/h)	5.0	39^10–11^	50^12–13^	59^14–15^	70^14–16^
Snow clearing (snow blower)	3.0	23^<10^	30^10–11^	35^10–11^	42^10–11^
Snow clearing (manual)	6.0	47^12–13^	60^14–16^	70^14–16^	84^15–17^
Lawn mowing (manual)	4.5	35^10–11^	45^12–13^	53^12–13^	63^14–16^
**Vigorous**					
Jogging 8.0 km/h	7.0	55^12–13^	80^14–16^	93^17–19^	>100^20^

** Activity of a certain energy cost might be perceived differently by people both as a function of age and of insufficient physical activity. Rating of perceived exertion (Borg scale): Very light <10; Light 10–11; Somewhat hard 12–13; Hard 14–16; Very hard; 17–19; Very, very hard 20. Table copied from NNR2012, page 204 (87).

## The WHO physical activity recommendation and energy requirement

The recommendations do not differ largely from the old concerning energy expenditure. The current WHO physical activity recommendations no longer refer to daily physical activity level (PAL). However, to calculate PAL the MET-value of different activities should be multiplied by time spent in the specific activity and divided by 24. For instance, an individual who sleeps 8 h (1 MET), engages 14 h in light intensity activity (2 METs), walks in moderate intensity for 2 h (5 METs) will have a PAL of 1.92.

## Physical activity guidelines in the Nordic and Baltic countries

In the Nordic and Baltic countries, there are no existing common recommendations for physical activity. Instead, each country created their own national guidelines according to their own protocol. Here, in this section, we give a synopsis of the status of country-specific recommendations. Table 4 lists the country-specific sources for information where the national guidelines can be found.

**Table 4:** Current physical activity and sedentary behavior guidelines in the Nordic and Baltic countries

Denmark	Reference or website
Children 0–5 years	0–1 years of age: Danish Health Authority (sst.dk) 1–4 years of age: Danish Health Authority (sst.dk)
Children 6–17 years (under 18)	Fysisk-aktivitet-–-håndbog-om-forebyggelse-og-behandling.ashx (sst.dk) (page 17)
Adults 18 years and above	Fysisk-aktivitet-–-håndbog-om-forebyggelse-og-behandling.ashx (sst.dk) (page 17)
SPECIFIC GUIDELINES	
Older adults 65+ years	Fysisk-aktivitet-–-håndbog-om-forebyggelse-og-behandling.ashx (sst.dk) (page 18)
Pregnant women	Fysisk-aktivitet-–-håndbog-om-forebyggelse-og-behandling.ashx (sst.dk) (page 18)
**Estonia**	
Children 0–5 years	https://intra.tai.ee/images/prints/documents/149019033869_eesti%20toitumis-%20ja%20liikumissoovitused.pdf (page 45)
Children 6–17 years (under 18)	https://intra.tai.ee/images/prints/documents/149019033869_eesti%20toitumis-%20ja%20liikumissoovitused.pdf (page 45)
Adults 18 years and above	https://intra.tai.ee/images/prints/documents/149019033869_eesti%20toitumis-%20ja%20liikumissoovitused.pdf (page 45) https://www.terviseinfo.ee/et/valdkonnad/liikumine/liikumispuramiid
**Finland**	
Children 0–5 years	http://urn.fi/URN:ISBN:978-952-263-413-9
Children 6–17 years (under 18)	http://urn.fi/URN:ISBN:978-952-263-861-8
Adults 18 years and above	https://ukkinstituutti.fi/en/products-services/physical-activity-recommendations/
SPECIFIC GUIDELINES	
Older adults 65+ years	https://ukkinstituutti.fi/en/products-services/physical-activity-recommendations/
Pregnant and postpartum women	https://ukkinstituutti.fi/en/products-services/physical-activity-recommendations/physical-activity-recommendation-during-pregnancy/ https://ukkinstituutti.fi/en/products-services/physical-activity-recommendations/weekly-physical-activity-recommendation-after-delivery/
Disabled persons	See children’s recommendations. Adults: https://ukkinstituutti.fi/en/products-services/physical-activity-recommendations/weekly-physical-activity-recommendation-for-adults-with-functional-limitations/
Chronic disease conditions	
**Greenland**	
Children 0–5 years	https://paarisa.gl/emner/det-gode-liv/fysisk-aktivitet/bevaegelse-for-de-mindste?sc_lang=da
Children 6–17 years (under 18)	https://paarisa.gl/emner/det-gode-liv/fysisk-aktivitet?sc_lang=da
Adults 18 years and above	https://paarisa.gl/emner/det-gode-liv/fysisk-aktivitet?sc_lang=da
SPECIFIC GUIDELINES	
Older adults 65+ years	https://paarisa.gl/emner/det-gode-liv/fysisk-aktivitet/bevaegelse-for-dig-der-er-over-65?sc_lang=da
Pregnant and postpartum women	https://paarisa.gl/emner/det-gode-liv/fysisk-aktivitet/bevaegelse-for-dig-der-er-gravid?sc_lang=da
**Iceland**	to be found at: https://island.is/hreyfing-radleggingar-landlaeknis
Children 0–5 years	
Children 6–17 years (under 18)	
Adults 18 years and above	
SPECIFIC GUIDELINES	
Older adults 65+ years	
Pregnant and postpartum women	
Disabled persons	
Chronic disease conditions	
**Latvia**	
Children 0–5 years	https://www.spkc.gov.lv/lv/fiziskas-aktivitates
Children 6–17 years (under 18)	https://www.spkc.gov.lv/lv/fiziskas-aktivitates
Adults 18 years and above	https://www.spkc.gov.lv/lv/fiziskas-aktivitates
**Lithuania**	
Children 0–5 years	http://www.smlpc.lt/media/image/Naujienoms/2017%20metai/Lankstukai/Bendrasis_Fizinis_aktyvumas_reko.pdf
Children 6–17 years (under 18)	http://www.smlpc.lt/media/image/Naujienoms/2017%20metai/Lankstukai/Bendrasis_Fizinis_aktyvumas_reko.pdf
Adults 18 years and above	http://www.smlpc.lt/media/image/Naujienoms/2017%20metai/Lankstukai/Bendrasis_Fizinis_aktyvumas_reko.pdf
**Norway**	
Children 0–5 years	https://www.helsedirektoratet.no/faglige-rad/fysisk-aktivitet-i-forebygging-og-behandling/barn-og-unge
Children 6–17 years (under 18)	https://www.helsedirektoratet.no/faglige-rad/fysisk-aktivitet-i-forebygging-og-behandling/barn-og-unge
Adults 18–64 years	https://www.helsedirektoratet.no/faglige-rad/fysisk-aktivitet-i-forebygging-og-behandling/voksne-og-eldre
SPECIFIC GUIDELINES	
Older adults 65+ years	https://www.helsedirektoratet.no/faglige-rad/fysisk-aktivitet-i-forebygging-og-behandling/voksne-og-eldre
Pregnant and postpartum women	https://www.helsedirektoratet.no/faglige-rad/fysisk-aktivitet-i-forebygging-og-behandling
**Sweden**	
Children 0–5 years	https://www.folkhalsomyndigheten.se/contentassets/106a679e1f6047eca88262bfdcbeb145/riktlinjer-fysisk-aktivitet-stillasittande.pdf
Children 6–17 years (under 18)	https://www.folkhalsomyndigheten.se/contentassets/106a679e1f6047eca88262bfdcbeb145/riktlinjer-fysisk-aktivitet-stillasittande.pdf
Adults 18 years and above	https://www.folkhalsomyndigheten.se/contentassets/106a679e1f6047eca88262bfdcbeb145/riktlinjer-fysisk-aktivitet-stillasittande.pdf
SPECIFIC GUIDELINES	
Older adults 65+ years	https://www.folkhalsomyndigheten.se/contentassets/106a679e1f6047eca88262bfdcbeb145/riktlinjer-fysisk-aktivitet-stillasittande.pdf
Pregnant and postpartum women	https://www.folkhalsomyndigheten.se/contentassets/106a679e1f6047eca88262bfdcbeb145/riktlinjer-fysisk-aktivitet-stillasittande.pdf

Each country has provided their own specific guidelines and no common guidelines exist. Some guidelines are a product of a scientific consultation process including public hearing and some are produced with less scientific contribution. Some are released by an NGO. Icelandic recommendations were not yet available at the time this article was published.

In general, most national recommendations are mirrored from the WHO recommendations, which, in turn, are based on epidemiologic evidence on the associations between physical activity and health. Some recommendations also state the frequency of activity and have slight differences in age group categories, but most refer to the duration, intensity or type of activity. Furthermore, some recommendations are created with the assistance of a national scientific advisory group and have included a review process of scientific evidence. Some recommendations rely on the existing evidence base and have replicated the WHO recommendations as they are. There are also some differences in the publishing organizations, where most recommendations are released by the Ministry of Health or subordinate agencies.

## How physically inactive are we?

Surveillance of physical activity levels has progressed substantially in the past decade including both standardized self-report questionnaires and different device-based methods. The challenge, however, is that there is low agreement between various instruments of self–reported physical activity and between subjective and objective assessment of physical activity (88). The use of device-based methods or wearable devices for population surveillance purpose have some concerns due to several methodological challenges, such as interpretation of data from acceleration into human behavior, location of devices, and inability to measure separate components of activity recommendation, mainly muscle-strengthening or balance training (1).

In pooled data analyses on self-reported physical activity – including data from 168 countries – global level of insufficiently active adults was estimated to be 27.5% in the adult population (89). The analyses also showed that the level of insufficient physical activity between 2001 and 2016 was stable. However, when looking at high-income countries the number of insufficiently active individuals has increased since 2001. The lowest level of physical inactivity was found in Finland (16.6%). Also, data from Finland suggest that sedentary time has been stable in the period 2007 to 2014 in the adult population (90).

Gender difference in surveillance data often suggests that men are physically more active than women, while device-based method reports higher levels for women (91). It may also be that men engage in higher intensity activities while women’s activity comprises more from moderate-to-low intensity physical activity (22). For differences across age groups, it is suggested that reaching the recommended levels of physical activity is more likely among the younger adults as compared to older adults (92).

Based on device measured physical activity harmonized analyses of more than 47 000 children and youth around Europe show the following: 29% of children and adolescents were sufficiently physically active, however, with substantial country differences in physical activity and sedentary time (93). For instance, physical activity level in the Nordic countries is higher compared to Southern European countries. Boys seem to be more active than girls throughout childhood and adolescence. Estimates from the study show that physical activity declines or levels off from the age of 6–7 years of age.

The prevalence of physical activity and meeting the activity guidelines varies across studies, which is due to different sampling frames, participation rates, assessment methods and analyzing techniques. For the Nordic countries, it is concluded that physical activity levels vary based on the chosen study and no conclusive statistics across age and gender groups are available. Taken together, independent of the methodological approach to assess the level of physical activity, there is a great potential to decrease sedentary time and increase physical activity in the population.
